# SPOT3D: Spatial positioning toolbox for head markers using 3D scans

**DOI:** 10.1038/s41598-019-49256-0

**Published:** 2019-09-06

**Authors:** Gaia Amaranta Taberna, Roberto Guarnieri, Dante Mantini

**Affiliations:** 1 Research Center for Motor Control and Neuroplasticity, KU Leuven, Leuven, Belgium; 2 Brain Imaging and Neural Dynamics Research Group, IRCCS San Camillo Hospital, Venice, Italy

**Keywords:** Neuroscience, Biomedical engineering

## Abstract

Recent studies have highlighted the importance of an accurate individual head model for reliably using high-density electroencephalography (hdEEG) as a brain imaging technique. Correct identification of sensor positions is fundamental for accurately estimating neural activity from hdEEG recordings. We previously introduced a method of automated localization and labelling of hdEEG sensors using an infrared colour-enhanced 3D scanner. Here, we describe an extension of this method, the spatial positioning toolbox for head markers using 3D scans (SPOT3D), which integrates a graphical user interface (GUI). This enables the correction of imprecisions in EEG sensor positioning and the inclusion of additional head markers. The toolbox was validated using 3D scan data collected in four participants wearing a 256-channel hdEEG cap. We quantified the misalignment between the 3D scan and the head shape, and errors in EEG sensor locations. We assessed these parameters after using the automated approach and after manually adjusting its results by means of the GUI. The GUI overcomes the main limitations of the automated method, yielding enhanced precision and reliability of head marker positioning.

## Introduction

Electroencephalography (EEG) is a non-invasive neuroimaging technique for measuring dynamic changes in electrical potentials over the scalp that are directly induced by neural activity. One of the most important factors for reliable source localization is the accuracy of the individual’s head model, including information on head structure extracted from magnetic resonance (MR) images, and on EEG sensor locations over the scalp^1–4^. Specifically, sensor positioning accuracy is mainly affected by the localization approach itself and the spatial registration of sensor positions in the coordinate system of the individual structural MR image.

EEG can be reliably used as a brain imaging technique when recordings are performed with more than 100 sensors. Indeed, high-density montages allow high-precision identification of neural sources and are therefore suitable for brain activity and connectivity analyses^5,6^. Commonly used techniques for collecting EEG sensor coordinates are electromagnetic or ultrasound digitization and photogrammetry^7–9^, whose spatial localization error ranges between 5 and 15 mm^1,10–13^. These widely used EEG positioning procedures are very time-consuming, particularly when dealing with high-density montages, and require extensive contribution by the operator^4,10,14^.

The precision of the spatial registration in individual MR space is closely related to the sensor positioning system used. Digitization and photogrammetry approaches provide only a few hundred points, corresponding to the EEG sensors embedded in the montage. However, these few data points cannot guarantee correct alignment with the whole head shape extracted from the MR image; indeed, the commonly used iterative closest point registration algorithm might converge to a local minimum of its cost function that does not correspond to the correct head-surface sensor configuration.

New localization techniques have been proposed, based on three-dimensional (3D) laser scanning^7,15^ or 3D infrared scanning^4,16^. In particular, the use of a colour-enhanced 3D scanner allows to obtain a point cloud of the whole surface of the individual’s head, thus achieving a more precise spatial registration in MR space. Thanks to the colour information and high resolution of the 3D scan, surface markers on the individual’s scalp can be localized with low positioning errors^4,16^. In our most recent work, we introduced an automated method for accurate spatial registration of a 3D scan to the structural MR image, as well as for detecting and labelling EEG sensors^17^.

The main limitations of the automated method are the lack of flexibility and the impossibility of user intervention. More specifically, since the accuracy of EEG sensor positioning and labelling depends on the number of false positives, we expect localization errors to increase with the number of sensors. In this study, we introduce a toolbox with a graphical user interface (GUI) that incorporates our automated spatial positioning pipeline. The name of the toolbox is SPOT3D, an acronym for *spatial positioning toolbox for head markers using 3D scans*. The GUI in SPOT3D enables the user to correct imprecisions in the spatial registration of the individual 3D scan to MR space, as well as in the identification of head marker positions. This enhances the positioning accuracy and the overall usability of the 3D scanning technique in comparison with our previous automated method^17^.

## Methods

SPOT3D is software written in the MATLAB environment (MathWorks, Natick, MA, US). The advantage of using this environment is that existing libraries and functions are easily included, and that portable, cross-platform software is obtained. The source code, together with some sample datasets and the software manual, is freely available for download at https://www.nitrc.org/projects/spot3d. The documentation specifies the software requirements and dependencies, and guides the user step by step through the whole processing pipeline.

### Software overview

**Figure 1:**
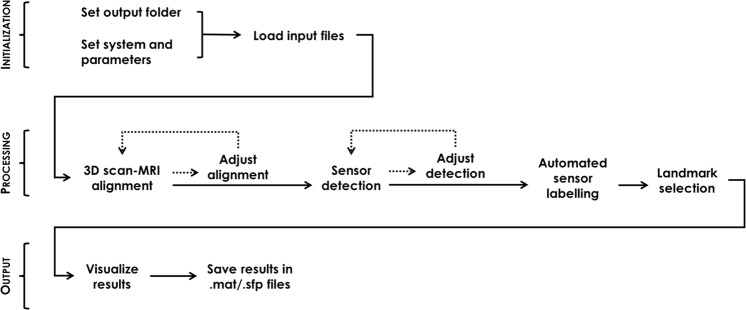
Workflow of the EEG sensor positioning system and use of the GUI. In the initialization phase, the user chooses the output folder, sets the system-specific parameters and loads the input files required. The 3D scan processing step comprises alignment in individual MR space, sensor detection on the aligned scan, automated sensor labelling and facial landmark selection. The editing tools accessible through the GUI allow the user to manually perform the 3D alignment in MR space as well as the sensor and landmark positioning from scratch, or to adjust them after automated processing. Finally, results are visualized and saved.

The SPOT3D user interface contains all the required functions for automated head-surface sensor detection. There is therefore no need for the operator to have experience in MATLAB programming, as the GUI offers a simplified, structured and user-friendly interface. The visual organization of the GUI is linear: first, the user initializes a new project and loads the required input files, and can then proceed with the 3D alignment and sensor detection steps. Both these modules can be performed manually by the user or automatically by running the related functions included in the software^17^; the automated output can also be adjusted manually before continuing the process. Once the user is satisfied with the adjustments, they can run the automated EEG sensor labelling function and manually select facial landmarks, if required. Lastly, they can visualize the final result and save it in the appropriate folder (Fig. 1). Below is a detailed description of the whole pipeline, including screen captures of the GUI. For further details on how to perform analyses of 3D scan data using SPOT3D, we refer to the manual distributed together with the software.

#### Initialization and DATA tab

**Figure 2:**
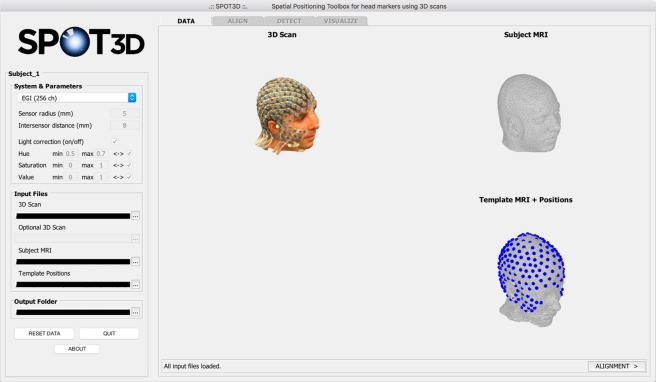
Screen capture of the SPOT3D interface, showing the *DATA* tab. On the left side of the window, the user can select system-specific parameters and load the input files, which are visualized on the right side. The dropdown menu in the *System & Parameters* panel presents different options: pre-set parameters for 128-channel or 256-channel EEG systems; *Custom* option, for user-defined parameters; *Empty (landmarks)* option, which requires no parameters.

The GUI is launched by typing the software name, *SPOT3D*, on the MATLAB command line; this opens the interface so that a new analysis can be started. The user selects the specific EEG montage to be considered, with its related parameters (see Section *System and parameters*), and the folder where the output files will be saved. They can then load the required input files: i) the 3D scan of the subject’s head; ii) an optional additional 3D scan for visualization purposes; (iii) the structural MR image of the subject; and iv) the system-specific template of 3D sensor positions. After loading, these files are visualized in the *DATA* tab, so that the user can easily check the inputs provided (Fig. 2).

As the main aim of SPOT3D is to detect head-surface sensors from 3D scanning data, it does not contain any tools for adjusting raw 3D scans, or any MRI pre-processing functions. Therefore, the user might need to adapt some input files before running the software. In particular, the bounding box of the subject’s 3D scan should include approximately the same space covered in the individual MR image and the MR image itself should be pre-processed to remove any intensity inhomogeneity artefacts.

System and parameters: In the *System & Parameters* panel, the user selects the specific sensor montage under consideration. The software includes pre-set parameters for some widely used EEG systems; the user can also set personalized parameters, by choosing the *Custom* option and manually inserting the required values. The system-specific parameters are related to the geometry of the montage – (i) *Sensor radius*, in mm; (ii) *Intersensor distance*, in mm – and to the colour of its sensors – (iii) *Hue*; (iv) *Saturation*; (v) *Value* (HSV space), each in the range [0, 1]. The three parameters related to sensor colour are expressed as minimum value, maximum value and internal/external interval between them to be considered. For example, given the *Hue* parameter and its minimum (*H*_*min*_) and maximum (*H*_*max*_) values, considering the internal or external interval means taking into account all the *H* values such that *H*_*min*_ ≤ *H* ≤ *H*_*max*_ or *(H* ≤ *H*_*min*_ ∨ *H* ≥ *H*_*max*_), respectively. The internal interval between values is considered by default. In addition to these parameters, the user can choose to perform some ambient light correction on the 3D scan before starting the sensor detection process (*Light correction on/off*)^17^.

When choosing the *Empty (landmarks)* option, no parameters or template sensor positions are required; after the alignment of the 3D scan in individual MR space, the GUI will let the user select the three facial landmarks – nasion (Nz), left pre-auricular point (LPA) and right pre-auricular point (RPA).

#### ALIGN tab

**Figure 3:**
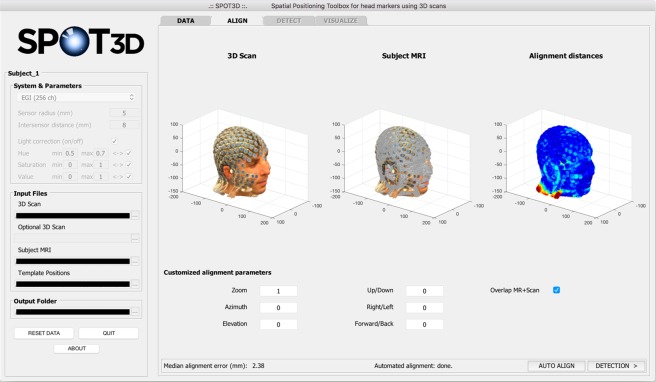
Screen capture of the SPOT3D interface, showing the *ALIGN* tab. In the upper part of the tab, the user can visualize from left to right, respectively: (i) the aligned individual 3D scan, (ii) the individual head shape extracted from the structural MR image (with or without overlap with the 3D scan) and (iii) a coloured map showing the alignment distances between (i) and (ii). Low errors are shown in blue, high errors in red. In the lower part of the tab, the user can set customized parameters to manually adjust the spatial alignment.

The second tab of SPOT3D, *ALIGN*, is designed for the co-registration of the subject’s 3D scan with the individual structural MR image. The user can choose to run the automated process^17^ or to perform manual alignment; in both cases, the result is shown in the GUI in terms of point-to-point and median alignment error. After automated alignment, the user can still adjust the output manually. Using the GUI, the alignment parameters – zoom, rotation and translation – can be customized and the result can be checked in the interface (Fig. 3). Specifically, each point of the aligned 3D scan, defined by a coordinate vector $${\boldsymbol{p}}=[\,\begin{array}{c}{p}_{x}\\ {p}_{y}\\ {p}_{z}\end{array}\,]$$, is mapped to a new point with coordinate vector $$\hat{{\boldsymbol{p}}}=[\,\begin{array}{c}{\hat{p}}_{x}\\ {\hat{p}}_{y}\\ {\hat{p}}_{z}\end{array}\,]$$, according to the following formula:

1 $$\hat{{\boldsymbol{p}}}=z\cdot R\cdot ({\boldsymbol{p}}-{{\boldsymbol{p}}}_{0})+{{\boldsymbol{p}}}_{0}+{\boldsymbol{t}},$$

In Eq. (1) each parameter acts as follows:

- $${{\boldsymbol{p}}}_{0}=[\,\begin{array}{c}{p}_{0x}\\ {p}_{0y}\\ {p}_{0z}\end{array}\,]$$: this is the center of mass of the whole 3D scan;

- *z* (zoom): this applies a factor of magnification (*z* > 1) or reduction (*z* < 1) to the 3D data;
- *R* (rotation): *Azimuth* and *Elevation* act on the rotation angles *az* and *el*, respectively, such that

$$R={R}_{az}\cdot {R}_{el},$$

where $${R}_{az}=[\,\begin{array}{ccc}\cos (az) & -\,\sin (az) & 0\\ \sin (az) & \cos (az) & 0\\ 0 & 0 & 1\end{array}\,]$$ and $${R}_{el}=[\,\begin{array}{ccc}\cos (el) & 0 & \sin (el)\\ 0 & 1 & 0\\ -\,\sin (el) & 0 & \cos (el)\end{array}\,]$$;

***t*** (translation): *Up/Down*, *Right/Left* and *Forward/Back* move the 3D data along the three Cartesian axes of the factors *ud*, *rl* and *fb*, respectively, so that $${\boldsymbol{t}}=[\,\begin{array}{c}rl\\ fb\\ ud\end{array}\,]$$.

#### DETECT tab

**Figure 4:**
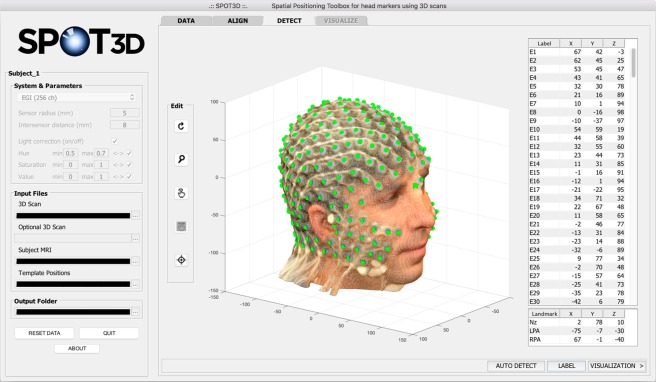
Screen capture of the SPOT3D interface, showing the *DETECT* tab. In the centre of the tab, the user can visualize the detected sensor positions; the Cartesian coordinates of each sensor are listed in the table on the right. Each position can be manually selected/corrected using the tools provided in the *Edit* panel on the left. The buttons, from top to bottom, allow the user to: rotate the scan, zoom in/out on the scan, select a sensor position, move an incorrectly detected position, and select facial landmarks. Automated labelling is performed by clicking on *LABEL*, in the bottom right corner of the tab; after that, the label assigned to each sensor is shown in the corresponding row in the table.

In the third step, sensor detection is performed on the 3D scan of the subject as obtained in the previous step. Again, the user can choose between running the automated process^17^ or manually selecting each sensor, using the tools provided in the *Edit* panel on the left side of this tab. After automated detection, the coordinates of each sensor can also be manually adjusted. The GUI allows the selected sensor positions to be visually inspected and for those incorrectly detected to be easily recognized and moved (Fig. 4). In particular, the *Edit* panel includes buttons that allow the user to: rotate the scan, zoom in/out on the scan, select a sensor position, move a wrongly detected position, and select the facial landmarks (Nz, LPA and RPA). Moreover, by clicking on *LABEL* in the lower part of the tab, the user can start the automated labelling of the sensors^17^.

#### VISUALIZE tab

**Figure 5:**
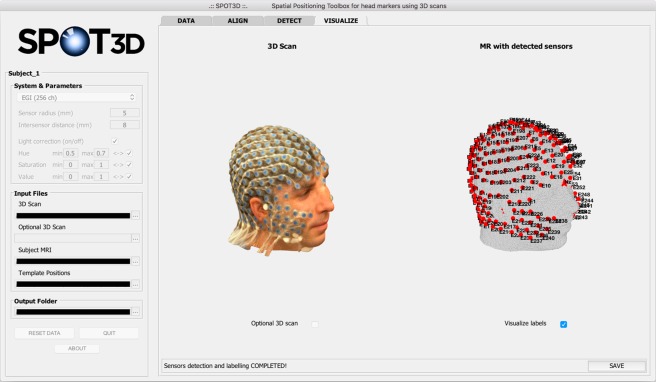
Screen capture of the SPOT3D interface, showing the *VISUALIZE* tab. This tab shows the individual 3D scan on the left and the head shape extracted from the structural MR image on the right, with the detected sensor positions visualized as red dots and, optionally, the facial landmarks visualized as red stars. The user can also choose to visualize the optional 3D scan and the label of each sensor/landmark. In the bottom right corner of the tab, the *SAVE* button allows the user to save the final sensor positioning results in .sfp and .mat formats.

At the end of the detection step, the software shows an overview of the result in the final tab, *VISUALIZE* (Fig. 5). To finalize the process, the user can save the results in the project folder. The list of sensor/landmark positions is saved as both.sfp and.mat files, and additional information on the individual 3D scan and on the MR-based head shape is stored as.mat file.

### Software validation

#### Dataset

We acquired data in four healthy volunteers aged 26–40 years. Each participant gave their prior written informed consent to the experimental procedures, which were approved by the Ethics Committee of KU Leuven. Participants gave also consent for publication of identifiable images. The experimental part of the study was performed in accordance with the relevant guidelines and regulations. It consisted of two sessions: in the first, a structural T1-weighted MR image was collected using a Philips Achieva 3.0T MRI system, with the same imaging parameters used in our previous work^17^; in the second, the participant wore a high-density EEG cap with 256 sensors (HydroCel Geodesic Sensor Net; Electrical Geodesics, Eugene, OR, USA), and two 3D scans of the participant’s head were acquired consecutively, in order to perform a test-retest analysis. We added blue circular markers (8 mm diameter) over the sensors to distinguish them from other support structures embedded in the EEG cap. The 3D scans were collected with a Structure Sensor camera (Occipital Inc., Boulder, CO, USA) mounted on an iPad Pro from Apple (Cupertino, CA, US); data acquisition and pre-processing were performed using the Skanect 3D software (Occipital Inc., Boulder, CO, USA).

Notably, we validated the GUI using a 256-electrode montage, for which electrode positioning is intrinsically more difficult than for the 128-electrode ones that were used in our previous study^17^. In this manner, it was easier to appreciate the added value of using a semi-automated instead of a completely automated approach.

#### Evaluation of method

To assess the accuracy of our method, we first manually selected and labelled all the EEG sensors on the 3D scans so that we could use these data for reference. Next, we started testing our user interface with the acquired data: we used the pre-set parameters for the specific EEG net analysed (Supplementary Table 1) and we ran the full pipeline.

Following the validation of the automated method proposed in our previous work^17^, we quantified: (1) the alignment error (AE) between the 3D scanning data and the MR image, calculated in correspondence of the 256 sensor positions; and (2) the intrinsic positioning error (PE) related to each sensor in the montage; (3) the time required for the spatial alignment and electrode positioning steps, respectively. Specifically, the intrinsic PE was obtained after a rigid co-registration of the detected sensor positions with the manual coordinates, to minimize the contribution of AE in our measures^12^. We calculated the AE and PE for each scan in each participant, so that we could also evaluate the test–retest deviation per participant (ΔAE and ΔPE, respectively). Additionally, to validate the editing tools provided in our GUI, we manually adjusted both the automated alignment output and the automated EEG sensor positioning output and quantified the new AE, ΔAE, PE and ΔPE for each scan in each participant. The computation time was measured using a computer with Mac-OS operating system, a 2.2 GHz Intel Core i7 processor and 16 GB RAM.

## Results

**Figure 6:**
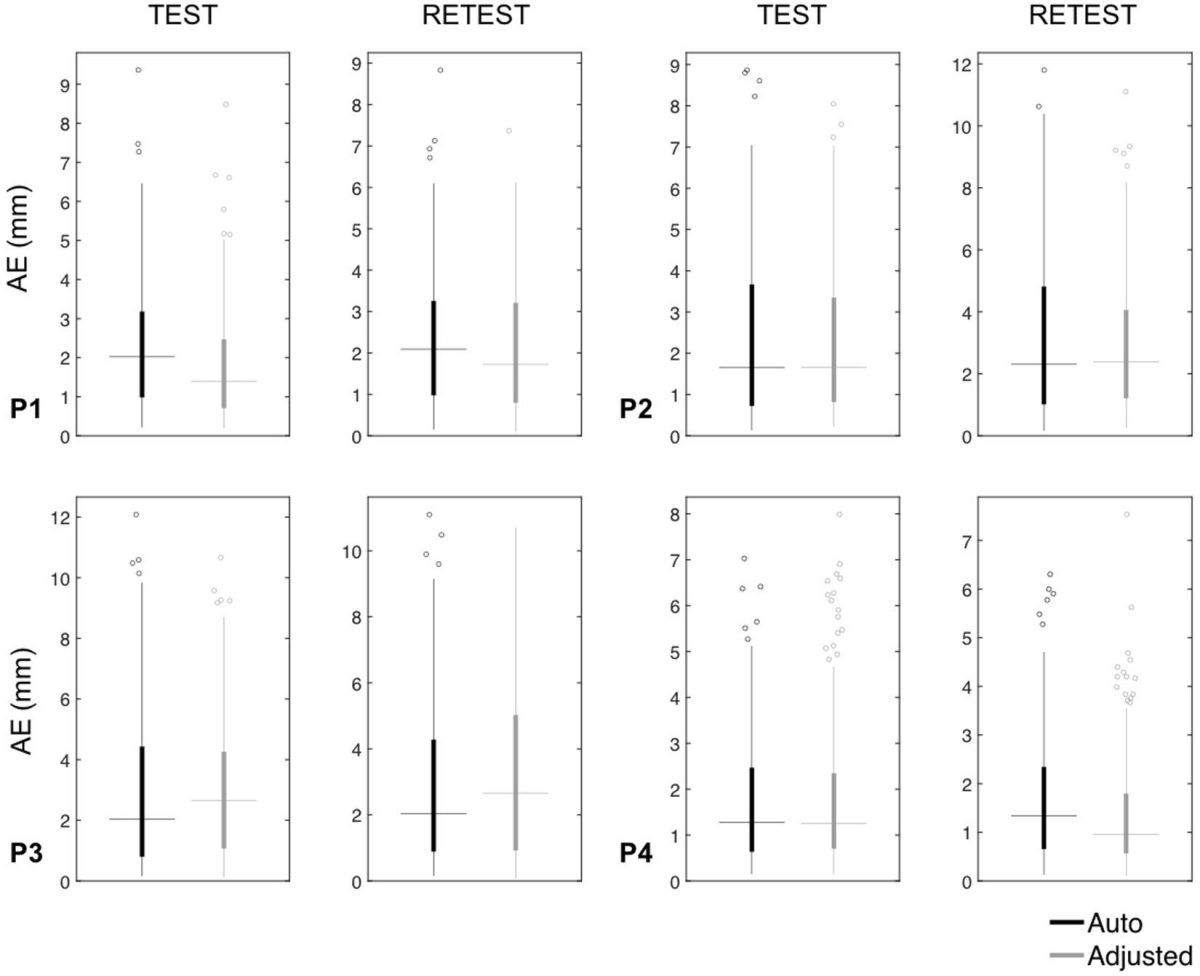
Alignment error (AE) for the spatial registration of the 3D scan to the individual MR image. Box plots show AE values obtained for each participant (P1–P4) after performing only the automated method or after manually adjusting the alignment output, for both test and retest acquisitions. The filled boxes include the values between 1st and 3rd quartile, the dots represent the outliers (i.e. the values outside 1.5 times the inter-quartile range below the 1st quartile and above the 3rd quartile), the horizontal lines indicate the median value and the vertical lines show the 1.5 interquartile range.

**Figure 7:**
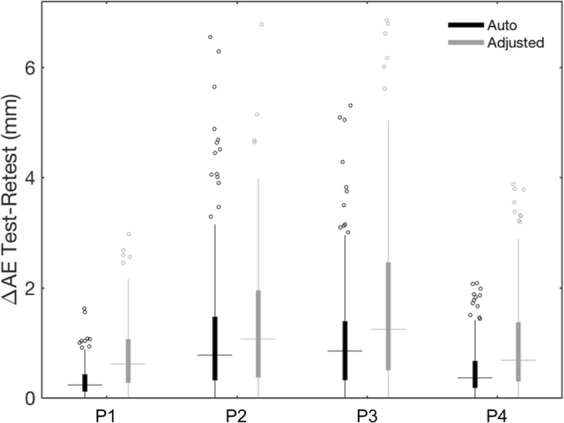
Test–retest analysis of the alignment error (AE) for registering the 3D scan to the MR image. Box plots show ΔAE values obtained for each participant (P1–P4), after performing only the automated method or after manually adjusting the alignment output. The filled boxes include the values between 1st and 3rd quartile, the dots represent the outliers (i.e. the values outside 1.5 times the inter-quartile range below the 1st quartile and above the 3rd quartile), the horizontal lines indicate the median value and the vertical lines show the 1.5 interquartile range.

Once the required input files are loaded for a new study using SPOT3D, the first step is to align the 3D scanning data with the head shape extracted from the structural MR image. This step is fundamental for moving the 3D scan into the coordinate space of the MR image and therefore minimizing co-registration errors between the two. Considering both scans for each participant, we obtained median AE values that never exceeded 2.65 mm and median ΔAE values not higher than 1.24 mm (Figs 6 and 7 and Supplementary Table 2). The average time required for the spatial alignment step was 358 and 514 seconds when run in automated and semi-automated modality, respectively.

**Figure 8:**
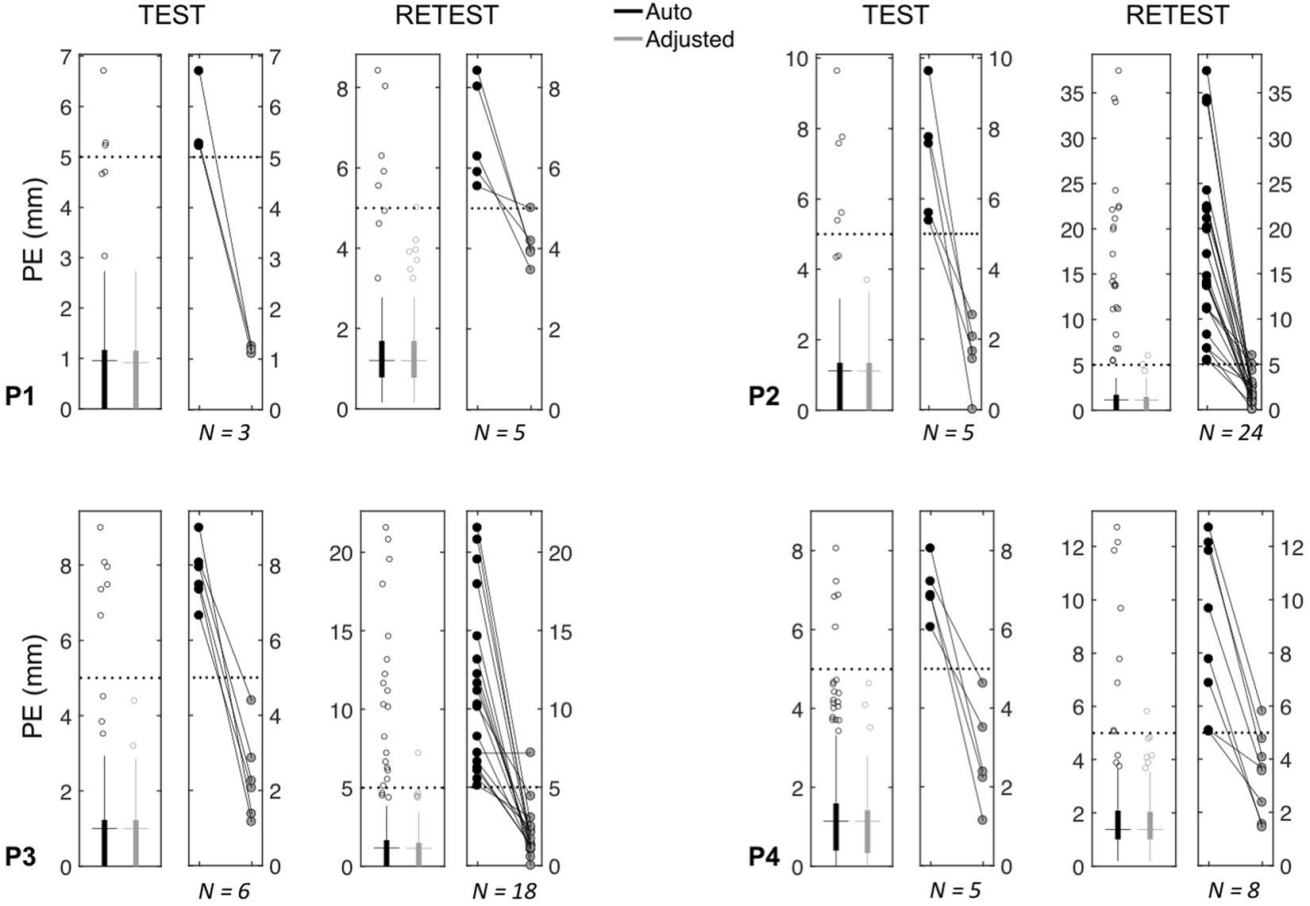
Positioning errors (PE) for the spatial positioning of EEG sensors. For each participant (P1-P4) and each scan (Test and Retest), the box plot on the left shows the PE values related to all sensors, either after performing only the automated method or after manually adjusting the detection output; the filled box includes the values between 1st and 3rd quartile, the dots represent the outliers (i.e. the values outside 1.5 times the inter-quartile range below the 1st quartile and above the 3rd quartile), the horizontal line indicates the median value and the vertical line shows the 1.5 interquartile range. The box plot on the right shows the PE of the sensor positions corrected using the editing tools in the GUI. The total number (N) of manually adjusted positions is reported under the related boxplot.

**Figure 9:**
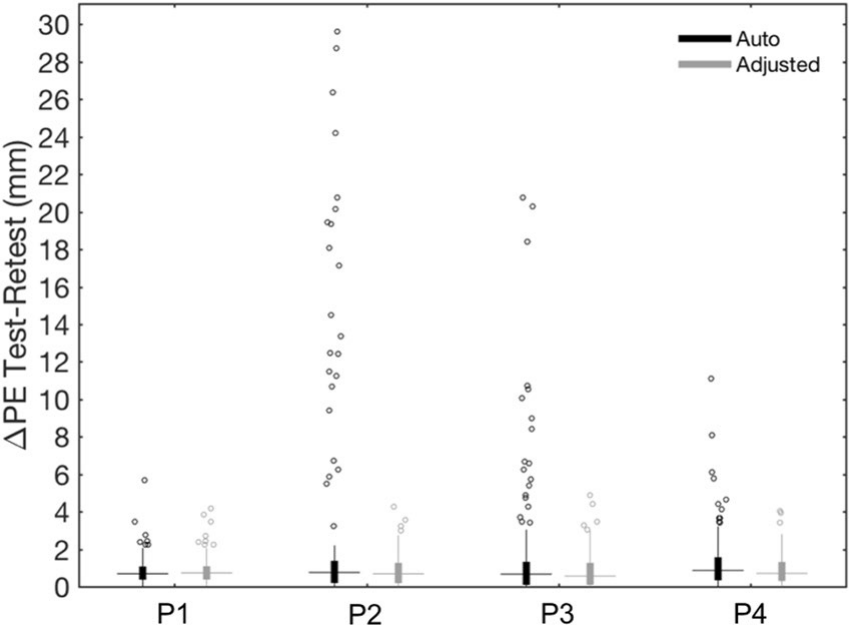
Test–retest analysis of the positioning error (PE) for the spatial localization of EEG sensors. The box plots show the ΔPE values obtained for each participant (P1–P4) after performing only the automated method or after manually adjusting the detection output. The filled boxes include the values between 1st and 3rd quartile, the dots represent the outliers (i.e. the values outside 1.5 times the inter-quartile range below the 1st quartile and above the 3rd quartile), the horizontal lines indicate the median value and the vertical lines show the 1.5 interquartile range.

After the alignment step, the interface guides the user to the sensor positioning on the co-registered 3D scan. Initially, we performed automated sensor detection; then, we visually inspected the output and used the editing tools in the GUI to correct the sensor positions and minimize localization error. Overall, we obtained an average median PE of 1.14 mm after the automated process and 1.12 mm after manual correction. The editing tools were very useful in terms of reducing the number of outliers. Notably, the average maximum PE decreased from 14.17 mm to 4.92 mm after manual adjustment, with respective maximum values going from 37.37 mm to 7.18 mm (Fig. 8 and Supplementary Table 3). The average median ΔPE also decreased from 0.75 mm to 0.67 mm after manual adjustment, and the average maximum ΔPE from 16.80 mm to 4.34 mm (Fig. 9 and Supplementary Table 3). The average time required for the electrode positioning step was 85 and 168 seconds when run in automated and semi-automated modality, respectively.

## Discussion

Accurate sensor localization is required for reliably reconstructing brain activity from EEG data. In a previous study, we presented an automated technique to detect and label the EEG sensors directly on structural MR images^12^. This method is not affected by the spatial registration problem in individual MR space and localizes sensors precisely; however, it can only be used when EEG data and MR images are collected during the same experimental session. The most widely used techniques for sensor positioning are still electromagnetic and ultrasound digitization, which have the considerable drawbacks of a strong dependence on environmental conditions, substantial operator involvement, and long procedure duration^8,13^.

New head-surface sensor positioning methods have recently been proposed, based on photogrammetry and 3D scanning. As the former requires cumbersome equipment^11,14^, an alternative is to use portable 3D scanners^7,15^. These methods are more accurate in detecting sensor positions, but are still prone to spatial registration errors^7^. To overcome these limitations, we have proposed an automated method for EEG sensor positioning and labelling, based on 3D scanning technology^17^. Notably, this automated method involves the spatial co-registration of the whole individual 3D scan with a head shape extracted from the individual structural MR image, with alignment errors comparable to those obtained with a manual approach^4,11^.

When precision in sensor positioning is fundamental, a manual approach might be preferred to an automated one. Indeed, despite the repeatability and ease of use of the latter, the former has the advantage of being more flexible, adaptable and user-oriented. Accordingly, we designed a GUI that guides the user through all the steps of 3D scan processing and allows them to manually adjust the results of the automated method or to manually perform each step from scratch. In this way, the overall precision of the sensor positioning process can be increased.

To assess the performance of the automated and semi-automated method by means of the GUI, we considered as reference positions those selected by an operator directly on the individual 3D scans^1,11^. Notably, this manual procedure still yields random errors that cannot be eliminated. When running the automated pipeline for 3D spatial co-registration of scanning data in MR space, we obtained median alignment errors of 1.27–2.31 mm, comparable with those found in previous studies^4,17^. We consider the automated process sufficiently precise to achieve correct spatial alignment in individual MR space; however, we believe that the GUI might still be useful, as it can give the user the possibility to fine-tune the result of the alignment step, before moving to the detection step.

The median positioning errors after automated sensor detection (range, 0.95–1.38 mm) were in line with our previous findings^17^ and with other studies based on photogrammetry and laser scanning^10,14,15^. We have shown that the use of the GUI can substantially improve spatial positioning: manual corrections decreased the maximum positioning error by 57.55% on average across participants, whereas the test–retest variability decreased by 63.06%. Moreover, a new feature implemented in the GUI is the possibility of manually selecting additional landmark points over the 3D scan of the participant, possibly extending the functionality of our software.

The computational time obtained for the automated alignment in MR space and the automated sensor detection, respectively, was consistent with our previous results^17^. Notably, the time required for manual adjustments in the spatial alignment and electrode positioning steps is largely dependent on the accuracy obtained using automated processing, the desired level of accuracy after correction, as well as on the level of experience of the user.

In conclusion, we have developed a GUI that complements the automated EEG sensor positioning approach we introduced in a previous study^17^. The GUI allows the user to perform manual adjustments, such that the precision of head marker positioning, and thereby neural activity reconstruction, can be enhanced^6^. We expect our interface to be suitable also for the detection of other visible markers on the individual’s scalp, such as magnetoencephalography (MEG) head-positioning coils^4,15^. Notably, 3D spatial registration in MR space is applicable to any 3D scan of the head, and sensor detection requires the tuning of only a few system-specific parameters. It should be considered, however, that the 3D scan of a participant not wearing a cap, for example in MEG acquisitions, could have a more irregular shape because of the participant’s hair. This might make automated alignment with the MR-derived head shape challenging. In this case, the user could take advantage of the manual editing tools provided in the GUI to achieve the desired 3D spatial alignment. As a future development, we aim to adapt our interface to work with any visible markers on the individual’s scalp.

## Supplementary information

Supplementary material

## Data Availability

The datasets generated and/or analysed for the present study are available from the corresponding author on reasonable request. SPOT3D toolbox, together with a sample dataset, is available for download at https://www.nitrc.org/projects/spot3d.
