# Supplementary material for: SPOT3D: Spatial positioning toolbox for head markers using 3D scans
Source: Sci Rep. 2019 Sep 6;9:12813. doi: 10.1038/s41598-019-49256-0 (PMC6731320; doi:10.1038/s41598-019-49256-0)
Supplement: Supplementary file 1 — Supplementary material [file 41598_2019_49256_MOESM1_ESM.pdf]

SPOT3D: Spatial positioning toolbox for head markers using 3D scans

Gaia Amaranta Taberna, Roberto Guarnieri, Dante Mantini

SUPPLEMENTARY MATERIAL

**Sensor radius (mm)**	5
**Intersensor distance (mm)**	8
**Light correction**	ON
**Colour range (HSV space)**	$0.5 \leq H \leq 0.7$<br>$0 \leq S \leq 1$<br>$0 \leq V \leq 1$

Supplementary Table 1: Parameters used for automated EEG sensor detection, specifically tuned for the HydroCel Geodesic Sensor Net (Electrical Geodesics, Eugene, OR, USA), with 256 embedded sensors.

		Median AE (mm)			MAD AE (mm)			Maximum AE (mm)		
		**Test**	**Retest**	**ΔAE**	**Test**	**Retest**	**ΔAE**	**Test**	**Retest**	**ΔAE**
**AUTO**	**P1**	2.02	2.09	0.23	1.12	1.12	0.15	9.35	8.82	1.62
	**P2**	1.65	2.31	0.77	1.10	1.62	0.54	8.85	11.78	6.54
	**P3**	2.03	2.04	0.85	1.43	1.36	0.53	12.06	11.08	5.31
	**P4**	1.27	1.44	0.36	0.75	0.81	0.22	7.01	6.17	2.08
**ADJ**	**P1**	1.40	1.72	0.61	0.78	1.04	0.38	8.47	7.35	2.97
	**P2**	1.66	2.38	1.07	1.11	1.34	0.75	8.03	11.08	6.77
	**P3**	2.64	2.65	1.24	1.60	1.92	0.82	10.65	10.70	6.85
	**P4**	1.26	0.96	0.68	0.65	0.48	0.48	7.98	7.52	3.88

Supplementary Table 2: Alignment error (AE) for co-registration to the MR image, obtained for test and retest acquisitions in each participant. AE values, expressed as the median, median absolute deviation (MAD) and maximum, are provided separately for the automated (AUTO) and manually adjusted (ADJ) alignment outputs. Test–retest deviation (ΔAE) values are also provided.

		Median PE (mm)			MAD PE (mm)			Maximum PE (mm)		
		**Test**	**Retest**	**ΔPE**	**Test**	**Retest**	**ΔPE**	**Test**	**Retest**	**ΔPE**
**AUTO**	**P1**	0.95	1.20	0.69	0.64	0.45	0.34	6.70	8.41	5.68
	**P2**	1.11	1.15	0.76	0.58	0.67	0.59	9.62	37.37	29.63
	**P3**	1.00	1.16	0.67	0.58	0.65	0.60	8.98	21.54	20.78
	**P4**	1.14	1.38	0.86	0.51	0.46	0.56	8.05	12.70	11.10
**ADJ**	**P1**	0.91	1.20	0.73	0.65	0.45	0.36	2.73	5.00	4.18
	**P2**	1.11	1.11	0.68	0.57	0.64	0.53	3.68	5.96	4.27
	**P3**	1.00	1.14	0.57	0.57	0.55	0.54	4.38	7.18	4.89
	**P4**	1.14	1.38	0.71	0.42	0.45	0.48	4.62	5.80	4.02

Supplementary Table 3: Positioning error (PE) for EEG sensor localization, obtained for test and retest acquisitions in each participant. PE values, expressed as the median, median absolute deviation (MAD) and maximum, are provided separately for the automated (AUTO) and manually adjusted (ADJ) detection outputs. Test–retest deviation (ΔPE) values are also provided.
